# Targeted eradication of EBV-positive cancer cells by CRISPR/dCas9-mediated EBV reactivation in combination with ganciclovir

**DOI:** 10.1128/mbio.00795-24

**Published:** 2024-06-14

**Authors:** Febri Gunawan Sugiokto, Renfeng Li

**Affiliations:** 1 Program in Microbiology and Immunology, University of Pittsburgh, Pittsburgh, Pennsylvania, USA; 2 Cancer Virology Program, Hillman Cancer Center, University of Pittsburgh Medical Center, Pittsburgh, Pennsylvania, USA; 3 Department of Microbiology and Molecular Genetics, University of Pittsburgh, Pittsburgh, Pennsylvania, USA; 4 Department of Oral and Craniofacial Molecular Biology, School of Dentistry, Virginia Commonwealth University, Richmond, Virginia, USA; 5 Philips Institute for Oral Health Research, School of Dentistry, Virginia Commonwealth University, Richmond, Virginia, USA; 6 Department of Microbiology and Immunology, School of Medicine, Virginia Commonwealth University, Richmond, Virginia, USA; 7 Massey Cancer Center, Virginia Commonwealth University, Richmond, Virginia, USA; Columbia University Medical Center, New York, New York, USA; Harvard Medical School, Brigham and Women's Hospital, Boston, Massachusetts, USA

**Keywords:** EBV, latency, reactivation, CRISPR/dCas9 activation, CMER, ganciclovir, targeted therapy, lymphoma, gastric cancer, nasopharyngeal carcinoma

## Abstract

Epstein-Barr virus (EBV) is a ubiquitous human tumor virus that establishes lifelong, persistent infections in B cells. The presence of EBV in cancer cells presents an opportunity to target these cells by reactivating the virus from latency. In this study, we developed a novel approach for EBV reactivation termed clustered regularly interspaced short palindromic repeats (CRISPR)/dCas9-mediated EBV reactivation (CMER) strategy. Using modified CRISPR-associated protein 9 (dCas9) fused with VP64, we designed 10 single guide RNAs (sgRNAs) to target and activate the EBV immediate-early gene promoter. In Akata Burkitt lymphoma cells, 9 out of 10 CMER sgRNAs effectively reactivated EBV. Among these, CMER sgRNA-5 triggered robust reactivation across various cell types, including lymphoma, gastric cancer, and nasopharyngeal carcinoma cells. Importantly, the combination of CMER and ganciclovir selectively eliminated EBV-positive cells, regardless of their cell origin. These findings indicate that targeted virus reactivation by CMER, combined with nucleoside analog therapy, holds promise for EBV-associated cancer treatment.

**IMPORTANCE:**

This study explores a novel strategy called clustered regularly interspaced short palindromic repeats (CRISPR)/dCas9-mediated Epstein-Barr virus (EBV) reactivation (CMER) to reactivate the Epstein-Barr virus in cancer cells. EBV is associated with various cancers, and reactivating EBV from latency offers a potential therapeutic strategy. We utilized an enzymatically inactive CRISPR-associated protein 9 (dCas9) fused with VP64 and designed 10 single guide RNAs to target the EBV immediate-early gene promoter. Nine of these sgRNAs effectively reactivated EBV in Burkitt lymphoma cells, with CMER sgRNA-5 demonstrating strong reactivation across different cancer cell types. Combining CMER with ganciclovir selectively eliminated EBV-positive cells, showing promise for EBV-associated cancer treatment.

## INTRODUCTION

Epstein-Barr Virus (EBV), also known as human herpesvirus 4, is one of the eight human herpesviruses that infects more than 90% of the adult population globally (1). EBV and Kaposi sarcoma-associated herpesvirus (KSHV) are part of the gamma-herpesvirus subgroup that is associated with various types of cancer (2). EBV infection of B cells normally establishes latency with limited viral gene expression. The virus occasionally reactivates during the B cell development process. In EBV-infected epithelial cells, the virus typically undergoes lytic replication. However, in EBV-associated epithelial cell cancers, the virus remains in a latent state (2).

In latently infected cancer cells, EBNA1 was shown to be a drug target (3). Upon reactivation, EBV-encoded protein kinase BGLF4 is expressed, which phosphorylates ganciclovir (GCV) into an active form (4). Phosphorylated GCV inhibits both viral and cellular DNA polymerases. The inhibition of viral DNA polymerase will block EBV replication and prevent the release of infectious virus. On the other hand, the inhibition of cellular DNA polymerase will result in cell death (5). Therefore, reactivating EBV from latency will provide an opportunity to selectively kill virus-infected cells.

In the past, various methods have been developed to trigger EBV reactivation and kill virus-infected cells with nucleoside analogs. Adenovirus vectors expressing EBV immediate-early (IE) genes (*ZTA/BZLF1* and *RTA/BRLF1*) have been used to induce reactivation in Burkitt lymphoma cells (5, 6). In addition, γ-irradiation, sodium butyrate, and chemotherapeutic agents (Bortezomib, cis-platinum, 5-fluorouracil, gemcitabine, and taxol) induce EBV reactivation in B cell or epithelial cell tumors (7–10). One potential concern is that these inducers lack specificity, as they not only target EBV-infected cells but also impact normal cells with toxicity.

Clustered regularly interspaced short palindromic repeats (CRISPR)/CRISPR-associated protein 9 (Cas9) has been extensively explored as a genome editor for mammalian cells (11, 12). In recent years, CRISPR/Cas9 has been adapted for gene activation or inhibition by fusing additional proteins and rendering the Cas9 catalytic site inactive (dCas9) (13, 14). VP64 is a transcriptional activator composed of four tandem copies of herpes simplex viral protein 16 (VP16) activation domain (amino acids 437–447: DALDDFDLDML). In 2013, the Joung group and the Gersbach group (15, 16) independently developed the dCas9-VP64 system for gene activation using the transient transfection method. The Jaenisch group (17) also developed multiple dCas9 expression vectors fused with different copies of VP16 for transient transfection. The Zhang group (18) later developed a dCas9-VP64 lentiviral vector with a blasticidin-resistant gene. The Ulitsky group created a similar dCas9-VP64 lentiviral vector with a puromycin-resistant gene. In this study, we employed the CRISPR/dCas9-VP64 vector carrying the puromycin-resistant gene to facilitate the straightforward selection of both B and epithelial cells for targeting the *ZTA* promoter, achieving the reactivation of EBV through a strategy termed CRISPR/dCas9-mediated EBV reactivation (CMER). The CMER approach robustly induces EBV reactivation across various EBV-positive cell types, including Burkitt lymphoma, gastric cancer, and nasopharyngeal carcinoma cells. Importantly, when combined with GCV, CMER selectively kills EBV-positive cells but not EBV-negative cells. This innovative strategy holds significant promise for further research and clinical applications.

## RESULTS

### Targeting EBV *ZTA* promoter by CRISPR/dCas9

**Fig 1:**
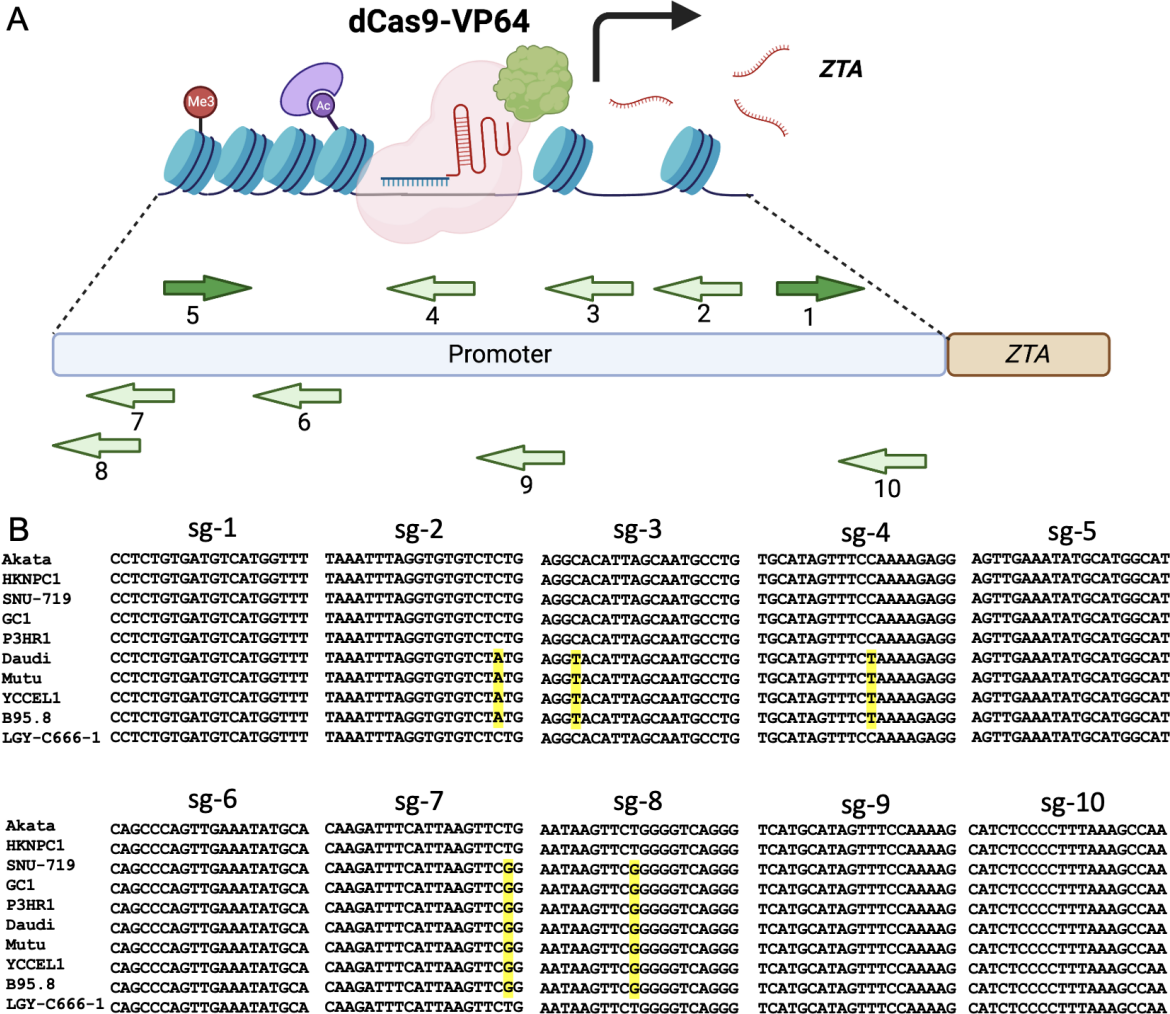
The design of sgRNA targeting EBV *ZTA* promoter. (**A**) Schematic representation of CRISPR/dCas9-VP64 targeting EBV *ZTA* promoter. The relative positions of sgRNA targeting sites were labeled as indicated. sgRNA-1 and sgRNA-5 (sg-1 and sg-5) target the sense strand, while the remaining sgRNAs target the anti-sense strand. (**B**) Sequence alignment of the sgRNA targeting sequences from 10 different EBV strains. Polymorphisms are highlighted in yellow. There are no polymorphisms in the protospacer adjacent motif sequences of the 10 sgRNAs.

The transition of EBV from latency to reactivation is regulated by the IE gene *ZTA* and, in some cases, *RTA* (19). Here, we focus on harnessing the power of the CRISPR/dCas9-VP64 gene activation system to specifically target the promoter of EBV *ZTA*. To do so, we strategically designed a series of single guide RNAs (sgRNAs) targeting the *ZTA* promoter within the Akata EBV+ genome (90,554–90,877 bp). We chose 10 unique sgRNA candidates from the array of designed sgRNAs to assess their potential for reactivating EBV (Fig. 1A).

To determine the sequence conservation of these sgRNA targeting sites, we analyzed 10 EBV strains derived from different cancer cells. Notably, we found that sequences targeted by sgRNAs such as sg-1, sg-5, sg-6, sg-9, and sg-10 are highly conserved, while those targeted by sg-2, sg-3, sg-4, sg-7, and sg-8 display single nucleotide polymorphisms (Fig. 1B). Therefore, sg-1, sg-5, sg-6, sg-9, and sg-10 are anticipated to target a wider range of EBV strains.

### CMER triggers EBV reactivation in B cells

**Fig 2:**
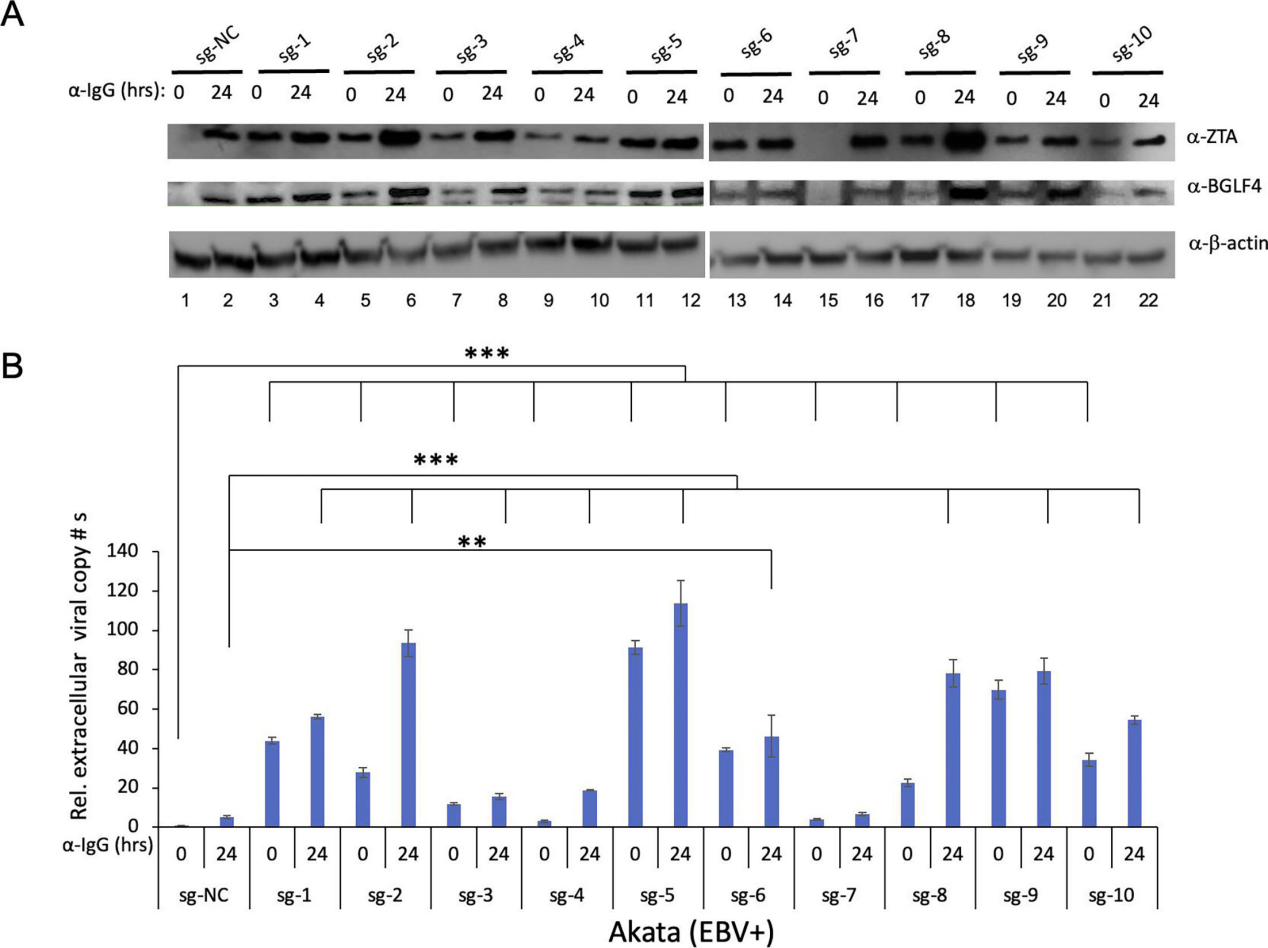
CMER promotes EBV reactivation in Akata (EBV+) Burkitt lymphoma cells. (**A**) Akata (EBV+) cells were used to create cell lines using lentivirus carrying dCas9-VP64 with control (sg-NC) and 10 *ZTA* promoter-targeting sgRNAs. The cells were uninduced (0 hour) or induced using anti-IgG for 24 hours. The expression levels of ZTA and BGLF4 were monitored by Western blot. β-Actin blot was included as loading control. (**B**) The relative extracellular EBV copy numbers were measured using quantitative polymerase chain reaction as described in Materials and Methods. The value of lane 1 was set as 1. Results from three biological replicates are presented. Error bars indicate the standard deviation (mean ± SD, **P < 0.01 and ***P < 0.001).

To test whether we can reactivate EBV using the CRISPR/dCas9-VP64 gene activation system, namely CMER, we cloned the 10 sgRNAs targeting *ZTA* promoter and a non-targeting control sgRNA into lentiviral system. Subsequently, we transduced Akata (EBV+) Burkitt lymphoma cells with lentivirus and established cell lines carrying individual sgRNA. The cells were either untreated or treated with anti-human IgG to induce lytic replication. We then monitored EBV ZTA expression levels in cells that were either untreated or treated with anti-human IgG to induce lytic replication. Interestingly, even without a lytic trigger, we found that ZTA is induced in cells carrying sgRNAs sg-1–sg-6 and sg-8–sg-10 (Fig. 2A, ZTA blot, lanes 3, 5, 7, 9, 11, 13, 17, 19, and 21). We also noticed that ZTA is not induced in cells carrying control sg-NC and sg-7 (Fig. 2A, ZTA blot, lanes 1 and 15). Because EBV protein kinase BGLF4 is the protein responsive for the phosphorylation of anti-viral nucleoside analogs, we also monitored its expression. Consistent with ZTA expression, BGLF4 was also induced in cells carrying sgRNAs sg-1–sg-6 and sg-8–sg-10 (Fig. 2A, BGLF4 blot, lanes 3, 5, 7, 9, 11, 13, 17, 19, and 21). The treatment with anti-human IgG induced the expression of ZTA and BGLF4 in all cell lines (Fig. 2A, lanes 2, 4, 6, 8, 10, 12, 14, 16, 18, 20, and 22).

To test whether EBV can complete its life cycle triggered by CMER, we measured the relative amount of EBV particles released to the culture media. We found that the extracellular viral copy numbers are significantly increased in all cells with sgRNAs targeting *ZTA* promoter, except sg-7 with minor increase (Fig. 2B). The treatment of cells with anti-human IgG further enhanced viral copy numbers in all cells. These results correlated well with the ZTA and BGLF4 expression. Excitingly, we noticed that sg-5 triggers the highest viral particle production (90-fold higher than control sg-NC), followed by sg-9 and sg-1 (Fig. 2B). These results together suggested that EBV can be reactivated by CMER, which provides an opportunity to target EBV for anti-cancer therapy.

**Fig 3:**
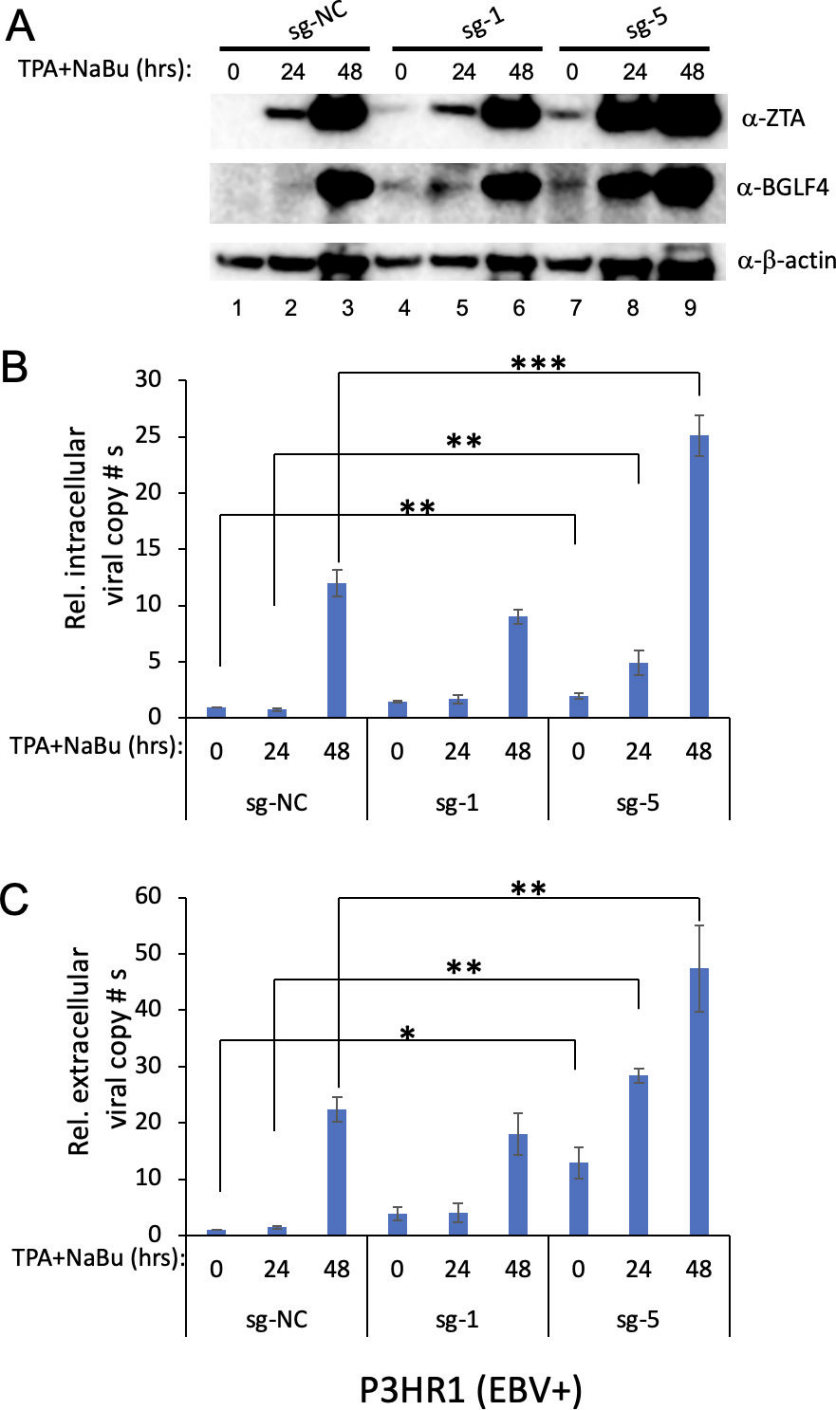
CMER triggers EBV reactivation in P3HR1 Burkitt lymphoma cells. (**A**) EBV-positive P3HR1 cells were used to create cell lines carrying dCas9-VP64 with control (sg-NC) and two *ZTA* promoter-targeting sgRNAs, sg-1 and sg-5. The cells were uninduced (0 hour) or induced using 12-O-tetradecanoylphorbol-13-acetate (TPA) and sodium butyrate (TPA/NaBu) for 24 and 48 hours. The expression levels of ZTA and BGLF4 were monitored by Western blot. β-Actin blot was included as loading control. (**B**) The relative intracellular EBV DNA copy numbers were measured using quantitative polymerase chain reaction (qPCR) as described in Materials and Methods. (**C**) The relative extracellular virion-derived DNA copy numbers were measured using qPCR as described in Materials and Methods. The value of lane 1 was set as 1. Results from three biological replicates are presented. Error bars indicate the standard deviation (mean ± SD, *P < 0.05; **P < 0.01; and ***P < 0.001).

The strong reactivation of EBV by CMER with most of the sgRNAs in Akata (EBV+) cells suggested that CMER can be applied to other EBV-positive cells. In addition to Akata (EBV+) cells, we tested EBV reactivation by CMER using another Burkitt lymphoma cell line, P3HR1. We selected two sgRNAs, sg-1 and sg-5, due to target conservation and reactivation efficiency to establish stable cell lines. We found that, without lytic induction, ZTA and BGLF4 are induced by CMER in cells carrying sg-5 and, to a lesser extent, sg-1 (Fig. 3A, lane 7 vs 4 and 1). After lytic induction, cells carrying sg-5 displayed the highest ZTA and BGLF4 expression (Fig. 3A, lanes 8 and 9), while sg-1-expressing cells had similar ZTA and BGLF4 expression as control cells (Fig. 3A, lanes 5 and 6 vs 2 and 3). These results suggest that CMER with sg-5 can trigger reactivation in P3HR1 cells. To further confirm these results, we measured intracellular EBV DNA copy numbers (Fig. 3B) and extracellular virion-derived DNA copy numbers (Fig. 3C). Consistently, we found that EBV copy numbers are much higher in sg-5-expressing P3HR1 cells than the control cells (Fig. 3B and C).

### CMER triggers EBV reactivation in epithelial cells

**Fig 4:**
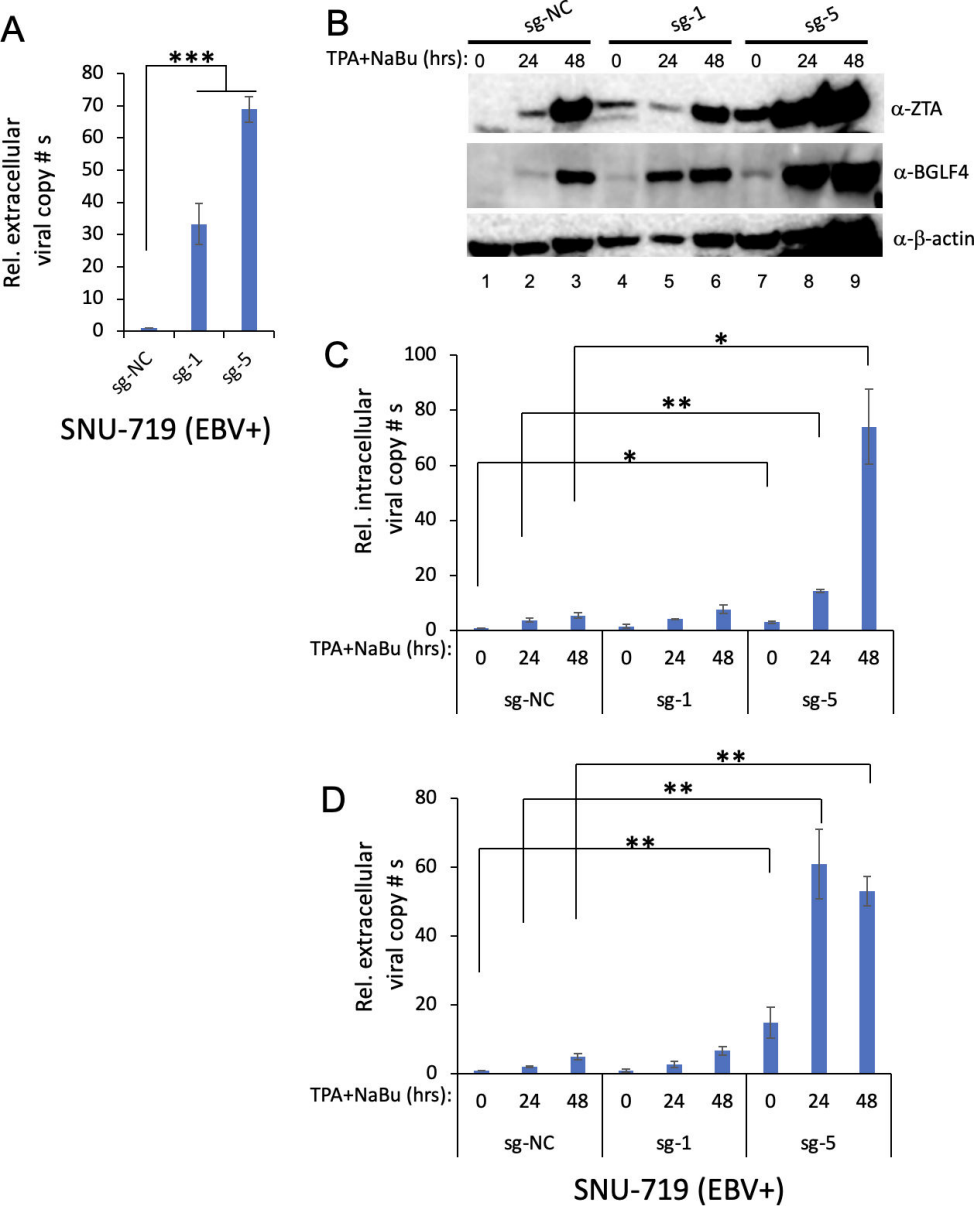
CMER triggers EBV reactivation in SNU-719 gastric cancer cells. (**A**) Lentiviruses carrying dCas9-VP64 with control (sg-NC), sg-1, or sg-5 sgRNAs were used to transduce EBV-positive SNU-719 cells. The relative EBV copy numbers secreted to the medium (72 hours post-lentiviral transduction) were measured using quantitative polymerase chain reaction (qPCR) as described in Materials and Methods. The cells were subsequently transferred to the T25 flask for cell line establishment. (**B**) SNU-719 cells carrying dCas9-VP64 with control (sg-NC), sg-1, or sg-5 sgRNAs were either uninduced (0 hour) or induced using TPA and sodium butyrate (TPA/NaBu) for 24 and 48 hours. The expression levels of ZTA and BGLF4 were monitored by Western blot. β-Actin blot was included as loading control. (**C**) The relative intracellular EBV DNA copy numbers were measured using qPCR as described in Materials and Methods. (**D**) The relative extracellular virion-derived DNA copy numbers were measured using qPCR as described in Materials and Methods. The value of lane 1 was set as 1. Results from three biological replicates are presented. Error bars indicate the standard deviation (mean ± SD, *P < 0.05 and **P < 0.01).

The process of EBV reactivation involves different signaling pathways in B cells and epithelial cells. To determine whether CMER could promote EBV reactivation in epithelial cancer cells, we first tested an EBV-positive gastric cancer cell line SNU-719. After the lentiviral transduction of SNU-719 cells, we found that cells carrying sg-1 and sg-5 start to detach from the plate (72 hours post-lentiviral transduction). We then collected the medium for examining extracellular viral copy numbers. The results showed that CMER strongly triggers EBV reactivation 3 days post-lentiviral transduction (Fig. 4A). Therefore, we initially had difficulty establishing a stable cell line carrying sg-1 and sg-5 as most of the cells failed to attach to the plate when we transferred the cells from the 6-well plate to T25 flask (72 hours post-lentiviral transduction). To get the cell lines, we kept culturing the cells until they reached high confluency (17 days for sg-1 and a month for sg-5). We then checked the EBV genes’ expression and found that cells carrying sg-1 and sg-5 but not sg-NC still express ZTA and BGLF4 even without lytic induction (Fig. 4B, lanes 4 and 7 vs 1). Lytic induction by 12-O-tetradecanoylphorbol-13-acetate (TPA) and sodium butyrate further enhanced ZTA and BGLF4 expression in sg-5-expressing cells, and to a lesser extent, sg-1-expressing cells (Fig. 4B, lanes 8 and 9 vs 5 and 6 vs 2 and 3). To validate these findings, we then quantified the intracellular EBV DNA copy numbers (Fig. 4C) as well as the extracellular virion-derived DNA copy numbers (Fig. 4D). Our results demonstrated significantly higher EBV copy numbers in SNU-719 cells expressing sg-5 compared to the control cells, regardless of whether lytic induction was present or not (Fig. 4C and D).

**Fig 5:**
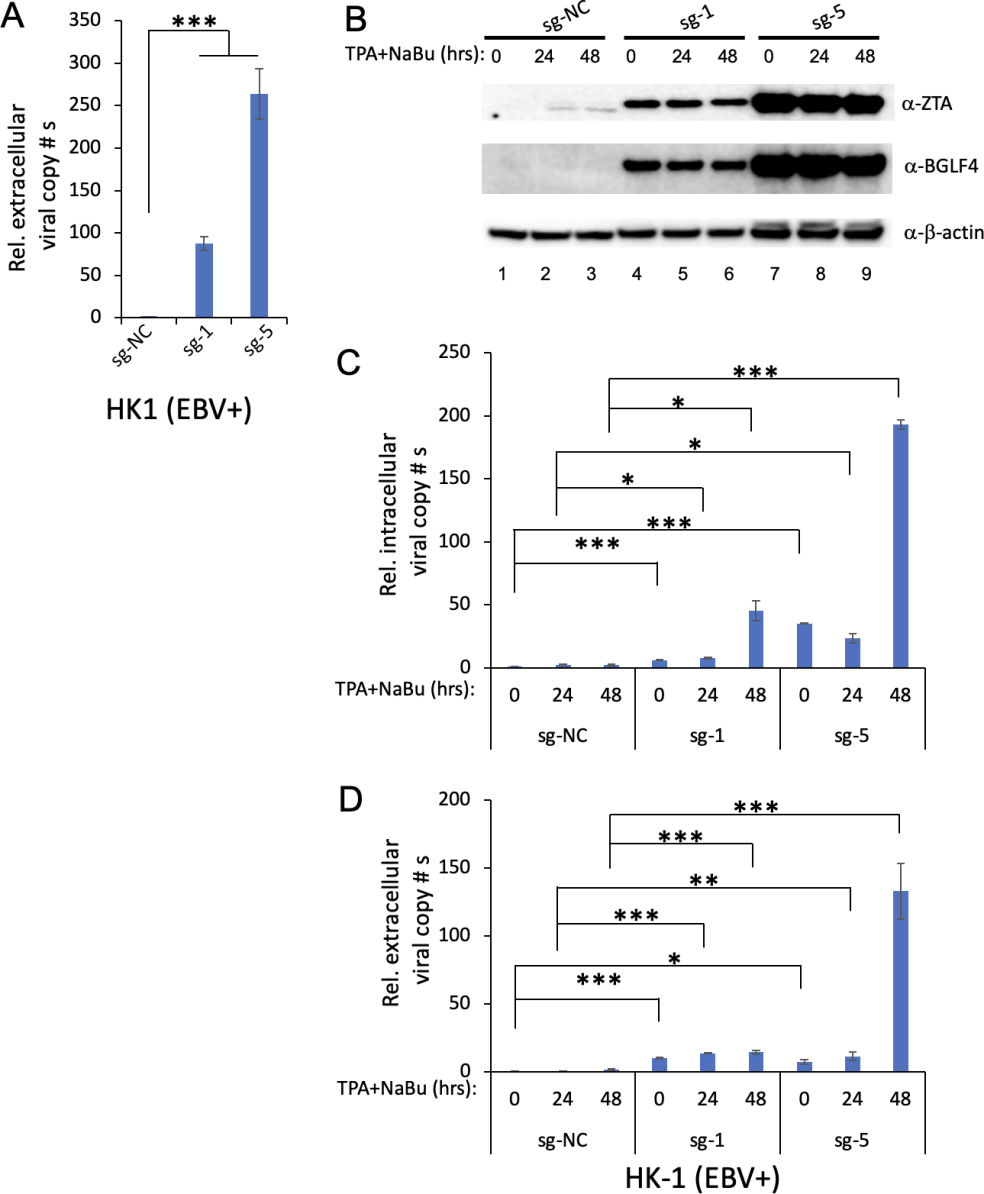
CMER triggers EBV reactivation in HK-1 (EBV+) nasopharyngeal carcinoma cells. (**A**) Lentiviruses carrying dCas9-VP64 with control (sg-NC), sg-1, or sg-5 sgRNAs were used to transduce HK-1 (EBV+) cells. The relative EBV copy numbers secreted to the medium (72 hours post-lentiviral transduction) were measured using quantitative polymerase chain reaction (qPCR) as described in Materials and Methods. The cells were subsequently transferred to the T25 flask for cell line establishment. (**B**) HK-1 (EBV+) cells carrying dCas9-VP64 with control (sg-NC), sg-1, or sg-5 sgRNAs were either uninduced (0 hour) or induced using TPA and sodium butyrate (TPA/NaBu) for 24 and 48 hours. The expression levels of ZTA and BGLF4 were monitored by Western blot. β-Actin blot was included as loading control. (**C**) The relative intracellular EBV DNA copy numbers were measured using qPCR as described in Materials and Methods. (**D**) The relative extracellular virion-derived DNA copy numbers were measured using qPCR as described in Materials and Methods. The value of lane 1 was set as 1. Results from three biological replicates are presented. Error bars indicate the standard deviation (mean ± SD, *P < 0.05; **P < 0.01; and ***P < 0.001).

To demonstrate the applicability of CMER in nasopharyngeal carcinoma cells, we utilized the HK-1 (EBV+) cell line. Similar to our observations in SNU-719 cells, we noticed that HK-1 (EBV+) cells exhibited cell death approximately 72 hours after lentivirus transduction, especially when transitioning from a 6-well plate to a T25 flask. At 72-hour post-lentiviral transduction, we also examined the released EBV copy numbers and noticed significant reactivation from the HK-1 (EBV+) cells by CMER with sg-1 and, more strongly, sg-5 (Fig. 5A).

It took around a month to establish a confluent HK-1 (EBV+) cell population with *ZTA* promoter-targeting sgRNAs, sg-1 and sg-5. After achieving cellular confluence, we induced the cells for reactivation for 24 and 48 hours. Intriguingly, we found that sg-1 and sg-5 expressed high levels of ZTA and BGLF4 compared to sg-NC, irrespective of lytic induction (Fig. 5B). We corroborated these results by measuring intracellular (Fig. 5C) and extracellular (Fig. 5D) viral copy numbers. The consistent findings demonstrated that sg-1 and, more strongly, sg-5 can spontaneously induce EBV reactivation in HK-1 (EBV+) cells, and lytic induction further enhanced EBV replication (Fig. 5C and D).

### CMER and GCV treatment selectively kills EBV-infected cells

For all EBV-positive cancer cell lines, we showed that CMER with sg-5 can consistently induce EBV reactivation regardless of lytic induction. This provides an opportunity to kill EBV-infected cells with nucleoside analogs.

GCV is recognized for its antiviral properties, particularly its ability to inhibit DNA synthesis. GCV enters cells in an inactive state and becomes phosphorylated by viral kinases, including EBV protein kinase BGLF4 (4, 20). Given our observations of active BGLF4 expression by CMER with sg-5 (Fig. 2A, 3A, 4B, and 5B), we sought to determine whether cells that have undergone EBV reactivation will be susceptible to GCV-induced cell death.

**Fig 6:**
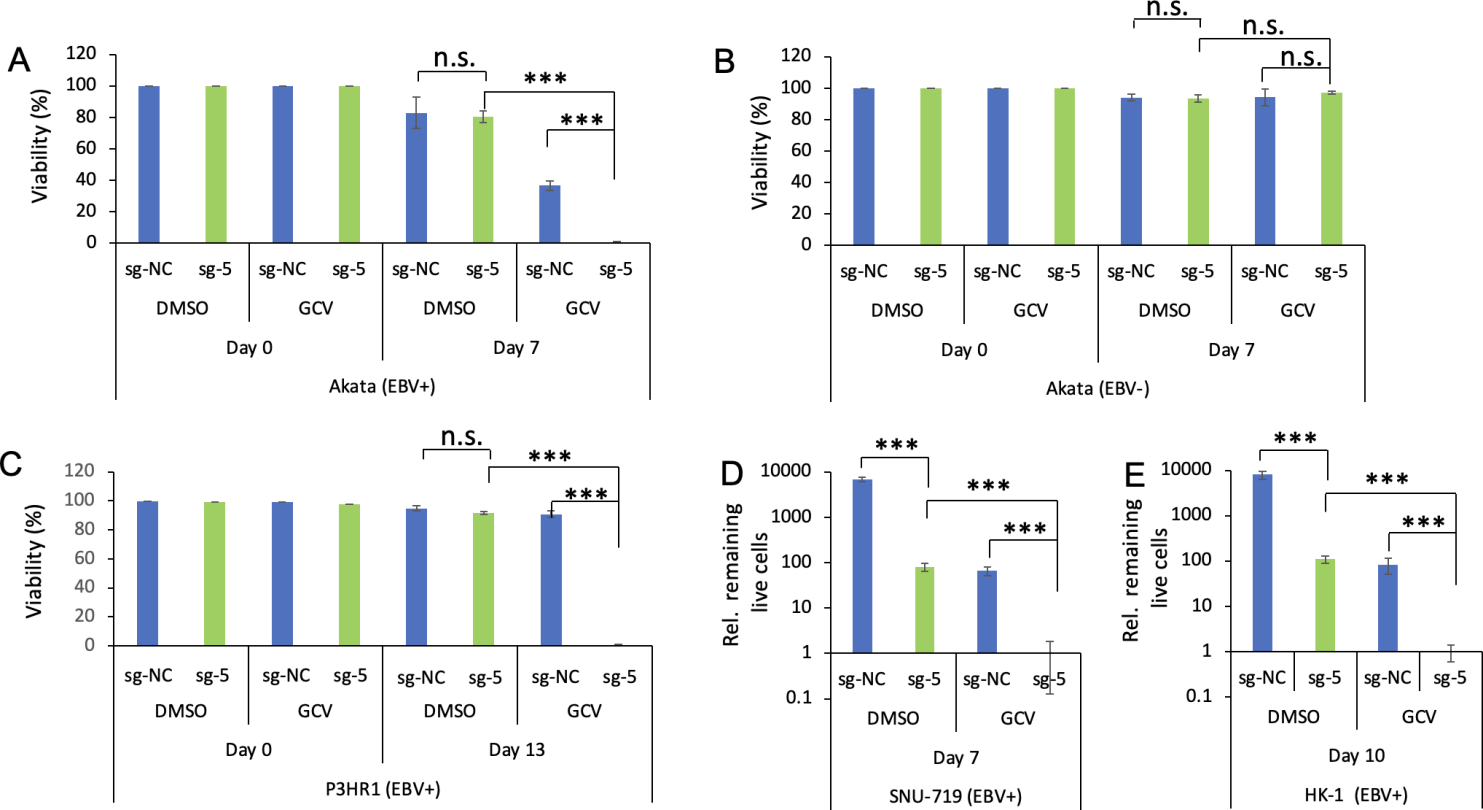
CMER and GCV treatment selectively kills EBV-infected cells. (**A**) Akata (EBV+) cells were transduced with lentiviruses containing dCas9-VP64 with sg-NC or sg-5. The cells were grown under puromycin selection together with DMSO or GCV for 7 days. The cell viability (live to total cells ratio) was measured as described in Materials and Methods. (**B**) Akata (EBV−) cells were transduced with lentiviruses containing dCas9-VP64 with sg-NC or sg-5. The cells were grown under puromycin selection together with DMSO or GCV for 7 days. The cell viability (live to total cells ratio) was measured as described in Materials and Methods. (**C**) P3HR1 (EBV+) cells were transduced with lentiviruses containing dCas9-VP64 with sg-NC or sg-5. The cells were grown under puromycin selection together with DMSO or GCV for 13 days. The cell viability was measured as described in Materials and Methods. (**D**) SNU-719 (EBV+) cells were transduced with lentiviruses containing dCas9-VP64 with sg-NC or sg-5. The cells were grown under puromycin selection together with DMSO or GCV for 7 days. The relative live cell numbers were counted. The number of sg-5-expressing cells treated with GCV was set as 1. (**E**) HK-1 (EBV+) cells were transduced with lentiviruses containing dCas9-VP64 with sg-NC or sg-5. The cells were grown under puromycin selection together with DMSO or GCV for 10 days. The relative live cell numbers were counted. The number of sg-5-expressing cells treated with GCV was set as 1. Results from three biological replicates are presented. Error bars indicate the standard deviation (mean ± SD, ***P < 0.001). n.s., no significance.

First, we used lentiviruses containing dCas9-VP64 with sg-NC or sg-5 to transduce Akata (EBV+) cells and then cultured the cells for 2 days. Subsequently, the cells were treated with puromycin and vehicle (DMSO) or GCV for 7 days. Notably, cells treated with DMSO displayed comparable viability between sg-NC and sg-5. Remarkably, when the cells were treated with GCV, sg-NC-carrying cells exhibited 40% viable cells, while sg-5-carrying cells almost died out (less than 1% viable cells) (Fig. 6A).

To rule out the potential off-target effects of sg-5 in inducing cell death, we also employed lentiviruses containing dCas9-VP64 with sg-NC or sg-5 to transduce Akata (EBV−) cells. These cells were subsequently treated with puromycin and DMSO or GCV for 7 days, and cell viability was assessed. Interestingly, we did not observe any differences in viability between the sg-NC- and sg-5-expressing cells treated with GCV (Fig. 6B). These findings strongly suggest that the combination of CMER and GCV has the potential to selectively eliminate EBV-positive cells, leaving EBV-negative cells unaffected.

To further demonstrate the utility of CMER and GCV combination in killing other EBV-positive B cells. We transduced P3HR1 (EBV+) cells with lentiviruses containing dCas9-VP64 with sg-NC or sg-5. Similarly, these P3HR1 cells were treated with GCV for a longer time (13 days) to observe cell-killing phenotype. We noticed that sg-NC-carrying cells exhibited more than 90% viable cells, while sg-5-carrying cells nearly reached extinction (less than 1% viable cells) (Fig. 6C).

The promising outcome achieved through the combination of CMER and GCV in eliminating EBV-positive B cells has motivated us to explore its potential application in EBV-positive epithelial cells. During the course of CMER and GCV treatment for adherent cell lines, we noticed that dying cells tend to detach from the culture plates. To monitor this process over multiple cell passages, we adopted a live cell counting approach. Notably, in the case of EBV-positive SNU-719 gastric cancer cells, we observed that sg-5-expressing cells grew much slower than sg-NC-expressing cells when treated with the vehicle (DMSO). Intriguingly, the addition of GCV resulted in the elimination of sg-5-expressing cells within 7 days of treatment (Fig. 6D). Similarly, the application of CMER and GCV to HK-1 (EBV+) nasopharyngeal cells led to the eradication of sg-5-expressing cells within 10 days of treatment (Fig. 6E).

**Fig 7:**
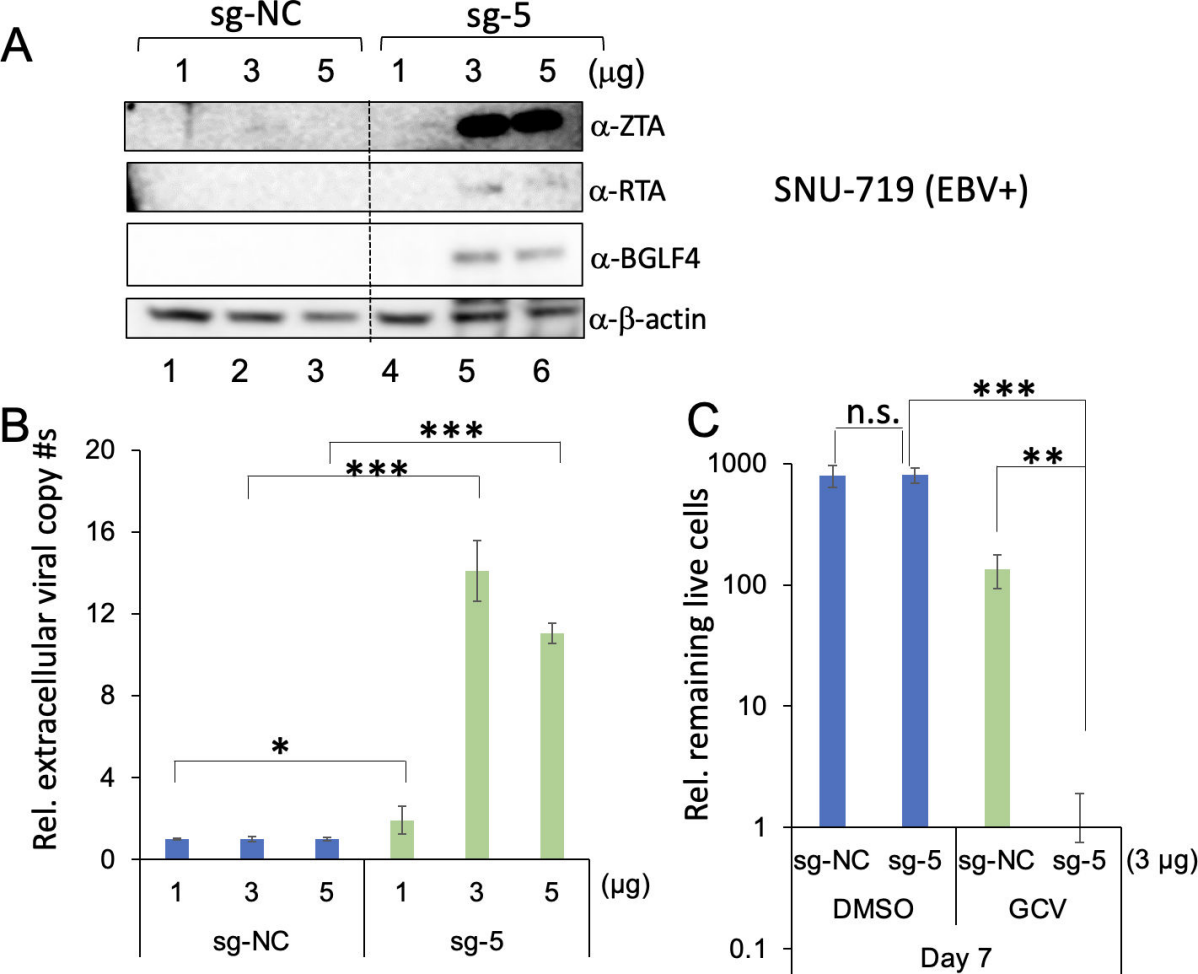
Delivery of CRISRP/dCas9-VP64 by transient transfection triggers EBV reactivation and subsequent cell death induced by GCV. (**A**) SNU-719 (EBV+) cells were transfected with pAC152-dual-dCas9VP64-sg-NC and pAC152-dual-dCas9VP64-sg-5 for 48 hours. The expression levels of ZTA, RTA, and BGLF4 were monitored by Western blot. β-Actin blot was included as loading controls. (**B**) The relative extracellular virion-derived DNA copy numbers were measured using quantitative polymerase chain reaction as described in Materials and Methods. The value of lane 1 was set as 1. (**C**) The transfected cells (corresponidng to lane 2 and lane 5 conditions in panel A) were treated with DMSO or GCV for 7 days. The relative live cell numbers were counted. The number of sg-5-expressing cells treated with GCV was set as 1. Results from three biological replicates are presented. Error bars indicate the standard deviation (mean ± SD, *P < 0.05; **P < 0.01; and ***P < 0.001). n.s., no significance.

In addition to lentiviral transduction, we also tested whether the transient transfection-based method could reactivate EBV. We created two plasmids for transient transfection assay, namely pAC152-dual-dCas9VP64-sg-NC and pAC152-dual-dCas9VP64-sg-5. We selected SNU-719 (EBV+) cells to test the reactivation by CMER because these cells can be transfected with high efficiency. We transfected these cells with different amounts of the plasmids and found that EBV is strongly reactivated at 48 hours post-transfection at a higher amount of the sg-5 plasmid (Fig. 7A and B). We also observed that the transfection of pAC152-dual-dCas9VP64-sg-5 and subsequent treatment with GCV kill SNU-719 (EBV+) cells (Fig. 7C). These results suggest that CRISPR/dCas9-VP64 can be delivered into cells via multiple methods to trigger EBV reactivation.

There are around 1% of GCV-treated sg-5-containing cells surviving at the end of our study (Fig. 6 and 7). There are several possibilities: first, those cells may eventually die after a longer incubation time. Second, these cells are resistant to GCV, which could be a concern for therapy. Third, there are small portion of EBV-negative cells that cannot be killed.

**Fig 8:**
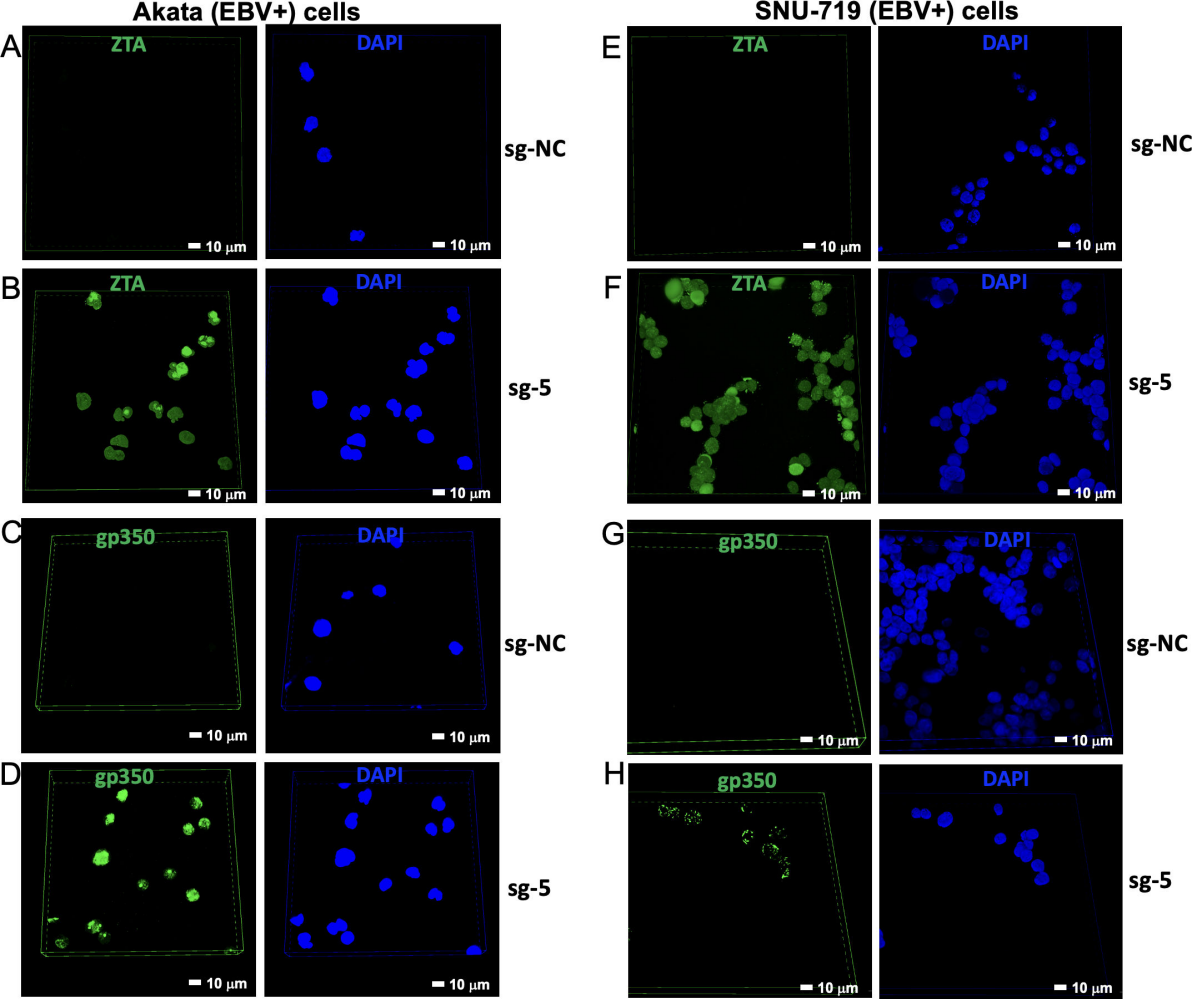
CMER reactivates EBV with 100% efficiency. Akata (EBV+) cells (**A–D**) and SNU-719 (EBV+) cells (**E–H**) carrying CRISPR/dCAS9-VP64-sgNC or sg-5 were blocked with 3% bovine serum albumin in PBS at room temperature for 1 hour and then incubated with anti-ZTA (**A, B, E, and F**) or anti-gp350/250 (**C, D, G, and H**) antibodies. Subsequently, the Alexa Fluor 488-labeled goat anti-mouse IgG antibody was added to the cells. Cell nuclei were stained with 4′,6-diamidino-2-phenylindole (DAPI) and visualized using a Nikon AXR microscope.

Although the combination of CMER and GCV can kill EBV-positive cells, it is a mystery that CMER itself cannot completely kill EBV-positive cells. To address this question, we performed immunofluorescence experiments targeting ZTA (CRISPR/dCas9 target gene product) and gp350 (late protein) (Fig. 8). Intriguingly, we found that both Akata (EBV+) cells (Fig. 8B and D) and SNU-719 (EBV+) cells (Fig. 8F and H) are reactivated by CRISPR/dCas9-sgRNA-5 with 100% efficiency while most of the cells are still viable at the time of our experiment. Based on these results, we reasoned that cancer cells may tolerate viral reactivation and replication. This also explains why GCV is needed to completely kill EBV-positive cells.

**Fig 9:**
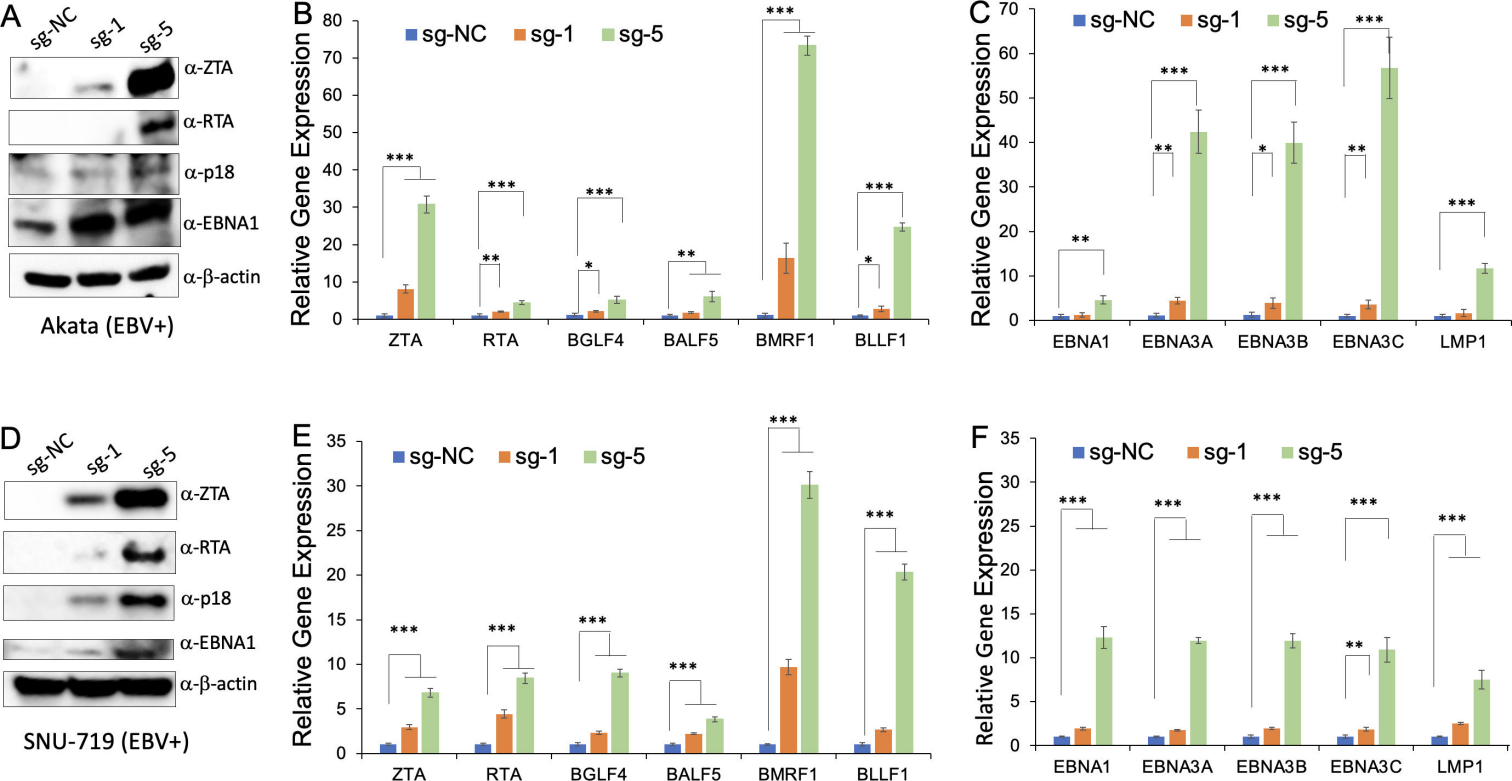
CMER triggers the expression of both lytic and latent genes. Akata (EBV+) cells (**A–C**) and SNU-719 (EBV+) cells (**D–F**) carrying CRISPR/dCAS9-VP64-sgNC or sg-5 were lysed to extract protein and RNA. (**A and D**) The expression levels of ZTA, RTA, p18, and EBNA1 were detected by Western blot. β-Actin blot was included as a loading control. (**B and E**) The mRNA levels of lytic genes (*ZTA*, *RTA*, *BGLF4*, *BALF5*, *BMRF1*, and *BLLF1*) were measured using RT-qPCR as described in Materials and Methods. (**C and F**) The mRNA levels of latent genes *(EBNA1*, *EBNA3A*, *EBNA3B*, and *LMP1*) were measured using RT-qPCR as described in Materials and Methods. Results from three biological replicates are presented. Error bars indicate the standard deviation (mean ± SD, *P < 0.05; **P < 0.01; and ***P < 0.001).

Our study suggested that CMER can trigger ZTA protein expression and EBV reactivation (Fig. 2 to 5). To test whether other lytic genes and latent genes are regulated by CMER, we monitored the levels of RTA (immediate-early protein), p18 (late protein), and EBNA1 (latent protein) by Western blot using Akata (EBV+) and SNU-719 (EBV+) cells. We observed that all these proteins were upregulated by CMER with sg-1 or sg-5 (Fig. 9A and D). We also measured the mRNA levels of several lytic and latent genes by reverse transcription (RT)-quantitative polymerase chain reaction (qPCR). We found that all these genes were upregulated by CMER with sg-1 and, more strongly, sg-5 regardless of cell types (Fig. 9B, C, E, and F). These results suggest that both latent and lytic genes are upregulated by CMER.

**Fig 10:**
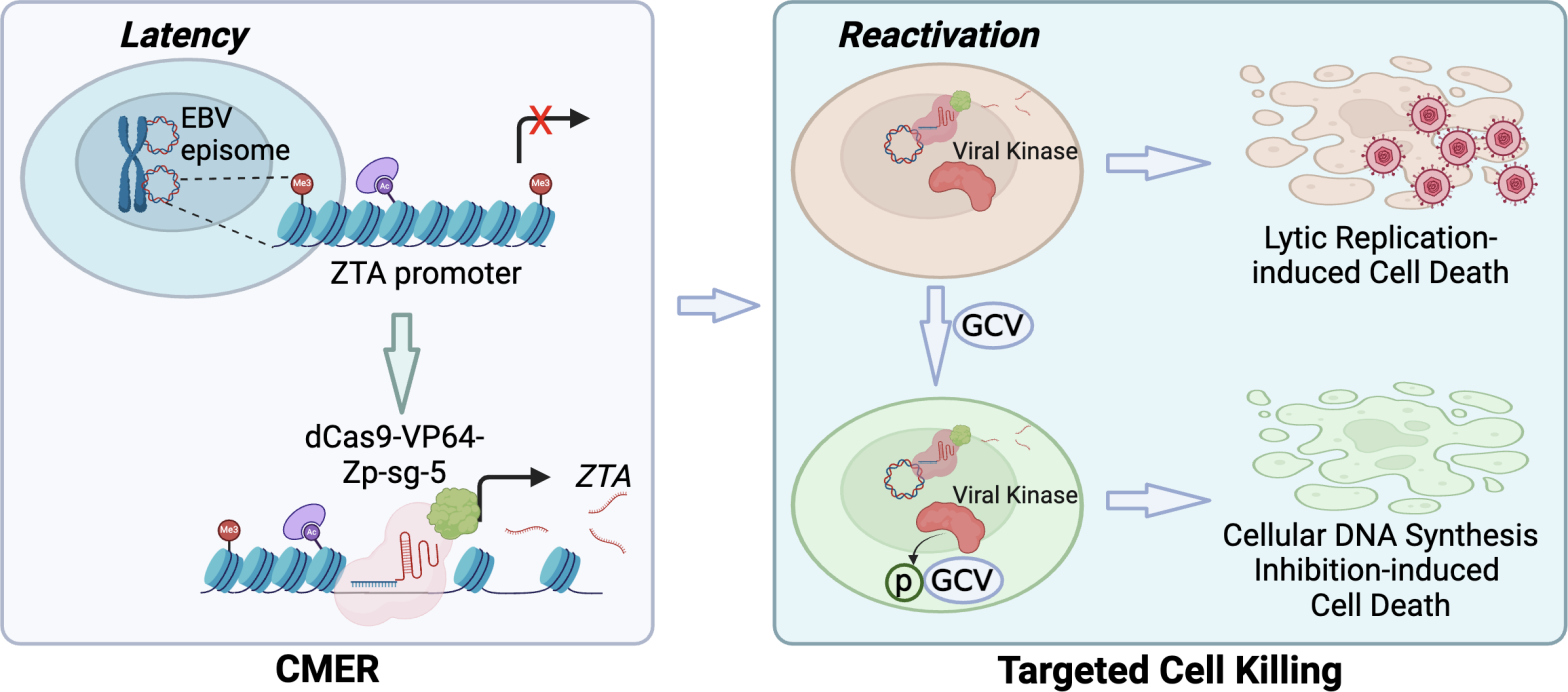
Model summarizing CMER in promoting cell death by EBV reactivation and GCV-mediated DNA synthesis inhibition. Lentiviral delivery or transfection of CRISPR/dCas9-VP64 and *ZTA* promoter targeting sgRNA (Zp-sg-5) promotes the expression of EBV ZTA and the downstream viral protein kinase (BGLF4). Strong lytic replication and the phosphorylation of nucleoside analog GCV by BGLF4 lead to the eradication of EBV-infected cancer cells.

Together, our study showed that CMER and GCV combination can selectively kill EBV-infected cancer cells regardless of cell type. In epithelial cells, we observed that CMER itself can lead to partial cell death due to strong lytic induction efficiency (Fig. 10).

## DISCUSSION

Since its application to edit the mammalian genome in 2013 (11, 12), the CRISPR/Cas9 technology has demonstrated remarkable potential across various fields. For example, CRISPR/Cas9 gene editing has been used to treat sickle cell disease (SCD) and transfusion-dependent β-thalassemia (TDT) (21). The autologous CD34+ cells were edited with CRISPR-Cas9 targeting the BCL11A enhancer to de-repress fetal hemoglobin expression. Ongoing clinical trials were initiated to evaluate the utility of this approach in treating SCD and TDT (ClinicalTrials.gov identifiers: NCT03655678 for TDT and NCT03745287 for SCD. On 8 December 2023, the U.S. Food and Drug Administration approved Casgevy and Lyfgenia as the first therapies utilizing CRISPR/Cas9 to treat SCD in patients 12 years of age and older. Recently, *in vivo* CRISPR-based therapeutic strategy, EBT-101, was used to treat HIV-1 infections in clinical trials (ClinicalTrials.gov identifiers: NCT05144386 for Phase 1/2 trial and NCT05143307 for long-term follow-up). This strategy utilizes an adeno-associated virus serotype 9 for intravenous administration to deliver CRISPR-Cas9 and guide RNAs, enabling a cleavage of multiple sites within the HIV-1 genome. This strategy facilitates the removal of substantial segments from the HIV-1 genome, reducing the likelihood of viral evasion.

In addition to CRISPR/Cas9-mediated gene editing, CRISPR/dCas9 fused with an activator or repressor can be used to enhance or repress gene expression, respectively (13). In this study, we developed a CMER strategy by harnessing a CRISPR/dCas9-VP64-mediated gene activation approach to induce EBV reactivation. Remarkably, we demonstrated that CMER with sg-5 can strongly reactivate EBV by enhancing ZTA expression, even though the number of EBV episome varies and the viral genomes are organized differently in B cells and epithelial cells (Fig. 2 to 5). Previously, CRISPR/dCas9 synergistic activation mediator was used to induce reactivation of HIV-1 latent reservoirs (22) with rare off-target effects (23). dCas9 fused to a destabilization domain and 12 copies of the VP16 activation domain (VP192) targeting KSHV ORF50 promoter also triggered an efficient KSHV lytic replication (24). These findings together with our results suggested that the CRISPR activation system can be utilized to reactivate latent viruses with therapeutic potential.

EBV reactivation is accompanied by caspase activation and subsequent cell death (25–29). This explains the rapid decline of SNU-719 (EBV+) and HK-1 (EBV+) cells shortly after lentivirus infection, coupled with the substantial release of extracellular EBV particles into the culture media (Fig. 4 and 5). Additionally, we observed that spontaneous EBV reactivation also results in elevated expression of viral protein kinase BGLF4 (Fig. 2 to 5). BGFL4 is the major kinase responsible for GCV phosphorylation (4). Phosphorylated GCV not only inhibits viral DNA replication but also cellular DNA replication, which leads to cell death (5). Motivated by this idea, we conducted a cell-killing assay combining CMER and GCV. Excitingly, we found that CMER and GCV combination kills EBV-positive Burkitt lymphoma cells without affecting cells without EBV (Fig. 6A through C). In addition to EBV-positive Burkitt lymphoma cells, we also demonstrated that CMER and GCV combination kills EBV-positive gastric cancer and nasopharyngeal carcinoma cells (Fig. 6D, E, and 7).

A previous study from Wang and Quake (30) utilized CRISPR/Cas9 approach to target EBV-latent genome. EBV-positive cells will undergo apoptosis if a large part of the EBV genome is deleted by CRISPR/Cas9. However, EBV-positive cancer cells contain multiple episomes (20–50 copies in Burkitt lymphoma cells and around 800 copies in EBV-positive gastric cancer cells, including SNU-719 [31]). Therefore, it is very challenging, if ever possible, to completely edit all EBV genomes. In contrast, our study takes advantage of EBV genomes in the cancer cells and utilizes the CRISPR/dCas9 activation system to target the *ZTA* promoter and reactivate EBV. Hence, a higher number of EBV genomes per cell make them more susceptible to targeting by CRISPR/dCas9. Indeed, our immunofluorescence analysis (Fig. 8) demonstrated that CMER with sgRNA-5 reaches 100% reactivation efficiency.

Although CMER showed partial killing activity, we consistently noticed that it could not kill all the cells by itself. Because CMER reactivates EBV with 100% efficiency while most of the cells are still viable, we reasoned that cancer cells may tolerate viral reactivation and replication. This explains why we also need GCV to completely kill EBV-positive cells.

Lytic induction by IgG crosslinking of B cell receptors or TPA/sodium butyrate promotes EBV reactivation and cell death. However, our previous studies have shown that cell death is likely compounded by the lytic triggers, which promote caspase activation (27, 29).

In addition to lytic genes, we also found that all latent genes are higher in cells carrying CRISPR/dCas9-VP64-sg1 and -sg5 (Fig. 9). According to previous studies, some latent genes, e.g., LMP1 and EBNA1, also contribute to EBV lytic replication process (32, 33). The contribution of other latent genes in lytic replication remains to be defined.

Because CMER does not target EBV-negative cells, we envision that it will have less adverse effects compared to chemotherapy drugs as lytic-inducing agents. Our strategy also has unique advantages compared to a previous method by overexpressing EBV IE genes (*ZTA* and *RTA*) as only EBV-positive cells will respond to CMER and express EBV IE genes. In addition to targeting EBV *ZTA* promoter, CRISPR activation can also be used to activate the expression of EBV protein kinase BGLF4 (4) to promote GCV phosphorylation without a full lytic cycle. For the potential clinical application of CMER, the toxicity of dCAS9-VP64 should also be carefully evaluated because the expression of an activator may induce basal expression of unwanted genes (34–37).

In summary, CMER provides a novel way to reactivate EBV with 100% efficiency without the need for other lytic-inducing agents. It will be interesting to test the percentage of the released viruses triggered by CMER that are infectious in the future. This not only provides a novel method to generate viruses from diverse sources for assessing EBV vaccine candidates but also, when combined with nucleoside analogs, lays a foundation for applications of the CRISPR/dCas9 activation system in clinical settings (Fig. 10). For delivering CRISPR/dCas9 *in vivo*, various vectors like adenoviral vectors, adeno-associated viral vectors, and lentiviral vectors could be employed (38). Adenoviral vectors have been used to deliver EBV *ZTA* and *RTA* with anti-tumor effects (6). The delivery of Cas9 protein through non-integrating lentiviral vectors has been used in treating SCD (39). However, a thorough assessment of the advantages and limitations of viral vectors is critical. Factors such as cell tropism, packaging capability, potential for viral integration into the host genome, and pre-existing immunities in the target population should be carefully evaluated. This ensures the selection of the most suitable vector for achieving the desired therapeutic outcomes while minimizing potential risks and complications.

## MATERIALS AND METHODS

### Cell culture and transfection

Akata (EBV+), Akata (EBV−), HK-1 (EBV+), SNU-719, and P3HR-1 cells were cultured in Roswell Park Memorial Institute medium (RPMI 1640) supplemented with 10% FBS (catalog no. 26140079, Thermo Fisher Scientific) at 37°C in a humidified 5% CO_2_ incubator (25, 27, 40–42). HK1 cells with the EBV recombinant Akata strain (courtesy of Dr. George Tsao, Hong Kong University) were supplemented with 800 µg/mL G418 in the culture medium (43–45). HEK293T cells were cultured in Dulbecco’s modified Eagle medium supplemented with 10% FBS in 5% CO_2_ at 37°C. To transfect plasmid DNA into the HEK293T cells, Lipofectamine 2000 was used following the manufacturer protocols (catalog no. 11668019, Life Technologies).

### Generation of DNA constructs and lentiviruses

Ten single guide RNAs targeting *ZTA/BZLF1* promoter were designed using CHOPCHOP (https://chopchop.cbu.uib.no/), and the primers were synthesized by Invitrogen. These 10 sgRNAs were cloned into the pLentiV2-dCas9-VP64 vector (gifts from Igor Ulitsky; Addgene: 141104). *Escherichia coli* Stbl3 strain was used to amplify the plasmids. Purified plasmids were co-transfected with psPAX2 and pMD2G (gifts from Didier Trono; Addgene: 12259 and 12260) into HEK293T for 48 hours to generate the lentivirus (27, 29).

The sgRNA primers for cloning are sg-1F: 5′-caccgaaaccatgacatcacagagg-3′; sg-1R: 5′-aaaccctctgtgatgtcatggtttc-3′; sg-2F: 5′-caccgtaaatttaggtgtgtctctg-3′; sg-2R: 5′-aaaccagagacacacctaaatttac-3′; sg-3F: 5′-caccgaggcacattagcaatgcctg-3′; sg-3R: 5′-aaaccaggcattgctaatgtgcctc-3′; sg-4F: 5′-caccgtgcatagtttccaaaagagg-3′; sg-4R: 5′-aaaccctcttttggaaactatgcac-3′; sg-5F: 5′-caccgatgccatgcatatttcaact-3′; sg-5R: 5′-aaacagttgaaatatgcatggcatc-3′; sg-6F: 5′-caccgcagcccagttgaaatatgca-3′; sg-6R: 5′-aaactgcatatttcaactgggctgc-3′; sg-7F: 5′-caccgcaagatttcattaagttctg-3′; sg-7R: 5′-aaaccagaacttaatgaaatcttgc-3′; sg-8F: 5′-caccgattaagttctggggtcaggg-3′; sg-8R: 5′-aaacccctgaccccagaacttaatc-3′; sg-9F: 5′-caccgtcatgcatagtttccaaaag-3′; sg-9R: 5′-aaaccttttggaaactatgcatgac-3′; sg-10F: 5′-caccgacatctcccctttaaagcca-3′; and sg-10R: 5′-aaactggctttaaaggggagatgtc-3′.

### Generation of stable cell line

Lentiviruses isolated from the HEK293T medium were used to infect the Akata (EBV+), HK-1 (EBV+), SNU-719, and P3HR-1 cells. Forty-eight hours post-transduction, the cells were cultured in the presence of puromycin (2 µg/mL) for cell line establishment.

### Immunoblotting

Cell lysis immunoblotting (Western blotting, WB) was performed as previously described (46). Briefly, whole cell lysates were separated using 4%–20% TGX gels (Bio-Rad). The proteins were then transferred to polyvinylidene difluoride membranes using a semidry transfer system. Membranes were blocked in 5% milk and probed with primary antibody and horseradish peroxidase-conjugated secondary antibodies.

Anti-ZTA(BZ1) antibody was purchased from Santa Cruz (catalog no. sc-53904, Santa Cruz), and Mouse anti-β-actin antibody was purchased from MP Biomedicals (catalog no. 691001, MP Biomedicals). Anti-BGLF4 antibody was a gift from Mei-Ru Chen (47). Anti-RTA antibody was from Argene (discontinued). Anti-p18 was purchased from Thermo Fisher (catalog no. PA1-73003, Thermo Fisher). Anti-EBNA1 (1EB12) antibody was purchased from Santa Cruz (catalog no. sc-81581, Santa Cruz).

### Lytic induction

Akata (EBV+) cells were treated with IgG (1:200; catalog no. 55087, MP Biomedicals) to induce lytic replication for up to 48 hours. To induce the EBV lytic cycle in P3HR-1 and SNU-719 cells, the cells were triggered with 12-O-tetradecanoylphorbol-13-acetate (TPA) (20 ng/mL; catalog no. NC9325685, Fisher Scientific) and sodium butyrate (3 mM; catalog no. 19137, Millipore) for up to 48 hours. For HK-1 (EBV+) cells, we used TPA at 40 ng/mL and sodium butyrate at 5 mM to trigger EBV reactivation.

### Quantification of EBV replication

To measure EBV replication, the levels of intracellular EBV DNA and virion-associated DNA were determined by quantitative polymerase chain reaction. For intracellular EBV DNA, total genomic DNA was extracted using a genomic DNA purification kit (catalog no. A1120, Promega) according to the manufacturer’s instructions. Extracellular viral DNA was extracted and measured as previously described (25). Briefly, the culture medium was treated with RQ1 DNase (catalog no. M6101, Promega) to remove free DNA at 37°C for 1 hour. The reaction was then deactivated by RQ1 DNase stop solution, followed by proteinase K and SDS treatment. The DNA was then purified by phenol-chloroform-isoamyl alcohol extraction. The relative viral DNA copy numbers were determined by qPCR using primers to the *BALF5* gene. The reference β-actin gene was used for data normalization as we described previously (48).

### Cell viability assay

Cells were infected with lentivirus for 48 hours and then selected under puromycin. Concurrently, GCV (10 µg/mL) was added to the culture medium. Fresh medium, puromycin, and GCV were replenished every 48 hours. The cells were harvested at various time points and subjected to the trypan blue exclusion assay (catalog no. 15250-061; Gibco).

For transient transfection assay, sg-NC and sg5 sequences were cloned into pAC152-dual-dCas9VP64-sgExpression vector (17) (a gift from Rudolf Jaenisch; Addgene: 48238). *Escherichia coli* DH5a was used to amplify and extract the plasmids. Purified plasmids were transfected to SNU-719 (3 × 10^5^ cells/mL) that have been grown overnight in RPMI containing 10% FBS using PEI-Max reagent (catalog no. 24765-100, Polysciences). The cells were harvested 48 hour after transfection. Expression of ZTA, RTA, BGLF4, and β-actin was detected using WB. For cell killing assay, after 48-hour transfection, the culture media were changed with fresh media containing GCV (10 µg/mL) and replenished every 48 hours. The cells were harvested 7 days later and subjected to the trypan blue exclusion assay.

### Immunofluorescence assay

Akata (EBV+) cells carrying CRISPR/dCas9-VP64-sg-NC and sg-5 were transferred to a 12-well plate containing poly-L-Lysine-treated coverslip and washed three times with phosphate-buffered saline (PBS). SNU-719 (EBV+) cells carrying CRISPR/dCas9-VP64-sg-NC and sg-5 were grown in UV-sterilized coverslip in a 12-well culture plate. The cells were fixed with cold methanol and washed three times with PBS. Cells were permeabilized with 0.5% Triton X-100 for 5 min and blocked with 3% bovine serum albumin for 1 hour at room temperature. The cells were washed three times with PBS and then incubated with anti-ZTA mouse monoclonal antibody (1:500) or anti-EBV MA-gp350/250 mouse antibody (1:500) (catalog no. MAB8183, Millipore Sigma) overnight at 4°C. The cells were washed three times with PBS and incubated with Alexa Fluor 488-conjugated goat anti-mouse IgG antibody (1:500) (catalog no. A11001, Invitrogen) for 1 hour at room temperature. After washing with PBS three times, the cell nuclei were stained with 4′,6-diamidino-2-phenylindole (DUO82040) and visualized using a Nikon AXR microscope.

### RNA isolation and RT-qPCR

Total RNA was extracted by Isolate II RNA minikit (Bioline) and analyzed by RT-qPCR with specific primers for EBV lytic and latent genes: ZTA-F: 5′-aggccagctaactgcctatc-3′, ZTA-R: 5′-tgattctgggttatgtcgga-3′; RTA-F: 5′-acactcccggctgtaaattc-3′, RTA-R: 5′-tggcttggaagactttctga-3′; BGLF4-F: 5′-ggcaatagaggcgatagagc-3′, BGLF4-R: 5′-tggtcctgactgattatggg-3′; BALF5-F: 5′-agtccttcttggctagtctgttgac-3′, BALF5-R: 5′-ctttggcgcggatcctc-3′; BMRF1-F: 5′- cgtgccaatcttgaggtttt-3′, BMRF1-R: 5′-cacccggggacttttatctt-3′; BLLF1-F: 5′-tactgcagtgggcatctctg-3′, BLLF1-R: 5′-tatggtggggtggtgtaggt-3′; EBNA1-F: 5′-ggacccggcccacaacctg-3′; EBNA1-R: 5′-ctcctgcccttcctcaccctcatc-3′; EBNA3A-F: 5′-ctaatggcctgtcgaatgg-3′, EBNA3A-R: 5′-tttcagcgcatcgacaca-3′; EBNA3B-F: 5′-ggatcgtcaccaccattgt-3′, EBNA3B-R: 5′-ggtgggatctgagcctattt-3′; EBNA3C-F: 5′-ggcacattgtcttccgtgtc-3′; EBNA3C-R: 5′-tacagactaccggcgagcat-3′; LMP1-F: 5′-tcctcctcttggggctactg-3′; and LMP1-R: 5′-tcatcactgtgtcgttgtcc-3′.

### Quantification and statistics

Statistical analysis employed a two-tailed Student’s *t*-test using Microsoft Excel software for comparison of two groups. A *P*-value less than 0.05 was considered statistically significant.
